# Starting a Swedish national quality registry for orthognathic surgery: a tool for auditing fundamentals of care

**DOI:** 10.1186/s12903-022-02568-6

**Published:** 2022-12-09

**Authors:** Mats Sjöström, Bodil Lund, Bo Sunzel, Martin Bengtsson, Mikael Magnusson, Lars Rasmusson

**Affiliations:** 1 Oral and Maxillofacial Surgery, Umeå University Hospital, Umeå, Sweden; 2 Department of Odontology, Umeå University, Umeå, Sweden; 3 Department of Dental Medicine, Karolinska Institute, Stockholm, Sweden; 4 Medical Unit of Plastic Surgery and Oral and Maxillofacial Surgery, Department for Oral and Maxillofacial Surgery and Jaw Orthopedics, Karolinska University Hospital, Stockholm, Sweden; 5 Dep Oral and Maxillofacial surgery Public Dental health Växjö, Malmö University, Malmö, Sweden; 6 Department of Clinical Sciences, Faculty of Medicine, Lund University, Lund, Sweden; 7 Department of Oral & Maxillofacial Surgery, Skåne University Hospital, Lund, Sweden; 8 Department of Specialist Dentistry, Oral and Maxillofacial Surgery, Colloseum and Smile AB, Stockholm, Sweden; 9 Department of Oral and Maxillofacial Surgery, The Sahlgrenska Academy and hospital, University of Gothenburg, Gothenburg, Sweden

**Keywords:** Quality registry, National patient registry, Orthognathic surgery, Launching patient registry

## Abstract

**Background:**

National quality registries (NQRs) provide open data for user-directed acquisition. National Quality Registry (NQR) data are often used to analyze the rates of treatment success and adverse events for studies that aim to improve treatment quality and patient satisfaction. Thus, NQRs promote the goal of achieving evidence-based therapies. However, the scientific literature seldom focuses on the complex process of initiating, designing, and implementing an NQR. Starting an NQR may be particularly challenging in a setting where specialized care is decentralized, such as orthognathic surgery in Sweden. The present study describes the initiation and early phases of a new NQR for orthognathic surgery in Sweden.

**Methods:**

The initial inventory phase included gaining knowledge on regulations, creating economic plans, and identifying pitfalls in existing NQRs. Next, a crude framework for the registry was achieved. Outcome measures were selected with a nation-wide questionnaire, followed by a Delphi-like process for selecting parameters to include in the NQR. Our inclusive process comprised a stepwise introduction, feedback-based modifications, and preparatory educational efforts. Descriptive data were collected, based on the first 2 years (2018–2019) of registry operation.

**Results:**

Two years after implementation, 862 patients that underwent 1320 procedures were registered. This number corresponded to a 91% coverage rate. Bimaxillary treatments predominated, and the most common were a Le Fort I osteotomy combined with a bilateral sagittal split osteotomy (*n* = 275). Reoperations were conducted in 32 patients (3.6%), and the rate of patient satisfaction was 95%.

**Conclusions:**

A National Quality Registry should preferentially be started and maintained by an appointed task force of active clinicians. A collaborative, transparent, inclusive process may be an important factor for achieving credibility and high coverage, particularly in a decentralized setting.

## Background

Currently, demands have increased for healthcare providers to ensure quality-audited, assured health care, evidence-based treatments that benefit the patient, and high patient satisfaction. Dentofacial deformities affect individuals in several ways. Asymmetries and discrepancies between the dental arches can affect masticatory function and speech; in addition, a distorted appearance can influence social life and self-esteem, which leads to low self-confidence [1]. Studies have shown that patients with dentofacial deformities were less satisfied with their appearance compared to a control group with normal features [2]. Moreover, they often experienced functional problems, such as difficulty chewing and speaking, and they have more symptoms at the temporomandibular joint and muscles and more headaches than individuals without dental deformities [3]. Thus, the primary motivation for seeking treatment is the desire to improve personal aesthetics, oral function, and self-confidence [4–6].

The combination of orthodontic treatment and orthognathic surgery aims to achieve harmonic dentofacial features and function [7]. Overall, orthognathic surgery comprises a group of surgical procedures that correct the relationship between the base of the skull and the dental arches (International Classification of Diseases [ICD]: 10 K07.1), between the two dental arches (ICD: 10 K.07.2), and other discrepancies in the facial skeleton. In the literature, studies have elucidated the indications [8, 9], assessment tools [10], surgical techniques [11–16], and timing for these surgical procedures [17]. Although these surgical procedures are associated with a wide spectrum of complications, the incidence of complications is reportedly low [18–20].

Orthognathic surgery evaluations are based on several outcome factors [21–23]. From the patient perspective, orthognathic surgery is demanding, time-consuming, and often expensive [24]. From a societal perspective, and for healthcare providers, the treatment is resource-demanding [25, 26]. All these factors motivate a careful analysis of whether the effort involved is justified by the treatment outcome.

Orthognathic surgery has been evaluated objectively by measuring postoperative dental occlusion, cephalometric parameter changes, facial photographs, and changes in temporomandibular disorders or functional impairments. Patient treatment satisfaction is correlated to aesthetics, functional issues, and psychosocial factors [27]. However, despite the large number of studies that have investigated several aspects of orthognathic surgery, there is a lack of solid evidence to support the value of orthognathic surgery. Österberg et al. [28] performed a literature search to evaluate the available knowledge in the field of oral and maxillofacial surgery. The results identified knowledge gaps in orthognathic surgery and concluded that well-conducted, large-scale clinical research was needed. To date, solid evidence is needed to support the value of orthognathic surgery; large-scale data are needed on patient satisfaction; and only rudimentary data are available to support treatment quality [28].

Modern healthcare institutions are expected to provide open data on the rates of success and adverse events. Additionally, decision-makers and funders require healthcare providers to provide reports on treatment results, safety, and efficacy. Recently, decision-makers have demanded that healthcare providers are evaluated based on quality registries. A quality registry is an information system that continously records event-based data on a all patients treated. Thus, a registry facilitates audits to ensure that the required quality of health care is performed. The general goal of a quality registry is to support clinical improvements and research aimed to ensure that patients receive the best possible care.

In Sweden, governmental support can be obtained for initiating a quality registry, provided that several predefined steps and requirements are fulfilled [29]. The Swedish Board of Health and Welfare performs yearly assessments to estimate the coverages of all national quality registries (NQRs) in Sweden. In 2019, NQRs covered 87% of all treatments [30]. Thus, NQRs provide a unique resource for collecting data on diagnoses, reasons for interventions, patient care, and treatment outcomes.

To improve the quality of orthognathic surgery, it is important to consider the patient ´s perspective [31, 32]. The better understanding a professional has of the patient’s perspective on treatment, the more they can improve the quality of the planned treatment [33]. In Sweden, with a population of around 10 million, 750–800 orthognathic surgical procedures are performed annually at 21 different hospitals [34]. In general, the incidence of complications is considered low. However, there is an unmet need to evaluate this patient group in terms of the diagnoses, the time spent in inpatient care, the types and extent of complications and the results of treatment.

Furthermore, we need to evaluate the benefit provided and patient safety, improve the quality of care, and secure equal care nationally. Further, it is essential to evaluate subjective patient impressions of facial appearance, jaw function, and oral health, from a quality-of-life perspective.

The present study aimed to describe the development and implementation of the first Swedish quality registry for orthognathic surgery (NROK) and report the novel data derived from this registry in 2018–2019.

## Methods

### Initiating, organizing, and designing the national quality registry

The decision to promote an NQR was made at the annual meeting of the Swedish Society of Oral and Maxillofacial Surgery in 2014. In 2015, a working group was assembled that comprised five oral and maxillofacial surgeons and one orthodontist, representing different geographic regions in the country. In an initial inventory phase, the group gained knowledge on regulations, initiated economic planning, and identified the pitfalls of existing NQRs. That phase produced a crude framework for the registry. The registry was nominated and the aims and goals of the registry were formulated (Table 1). At this stage, a crucial factor for success was the necessity to integrate registration into the everyday routine of the busy clinician. Based on previous experience with other NQRs, a user-friendly web-based platform was created, with a registration form and a 5-min time limit for entering data. This process was partly supported by one of six Swedish Registry Centers, which provided the adjustable digital platform, Stratum (Registercentrum Västra Götaland, Gothenburg, Sweden). Stratum ensured digital stability, high safety, compliance with General Data Protection Regulations, and all other legal aspects of a patient registry platform.

**Table 1:** The predefined denotation, acronym, aim, and goal of the patient registry and current website directory

Register name	Swedish quality register for orthognathic surgery (English) Nationella registret för ortognatkirurgi (Swedish)
**Abbreviation**	NROK
**Aims**	• *To quality assure the orthognathic care as well as continuously document and monitor its patient benefit and safety.* • *To assure equal care and form basis for consensus regarding indications and contribute to development of orthognathic surgery.* • *To support research within the field and thereby fill knowledge gaps that previously have been identified.*
**Goals**	• Registry coverage rate of total number of operations: 90% • Patient satisfactory: 90% • Complications: < 10%
**Website**	https://nrok.registercentrum.se

An important factor in accomplishing high coverage was the relevance of the data for primarily affiliated surgeons, users (patients), and funders and decision makers. To achieve high relevance, we implemented a collaborative, transparent, inclusive process for selecting outcome measures by sending a nation-wide web-based questionnaire to all active oral and maxillofacial surgeons in Sweden. All suggested outcome measures were scrutinized by the working group with a Delphi-like method. This step of development had the predefined goal of full agreement within the working group. Iterative, open discussions were conducted with controlled feed-back. When a full consensus was achieved in the working group, the outcome measures were packaged in hard copies, and distributed for testing at the six largest Oral and Maxillofacial Clinics in Sweden for a 2-month period. The completed questionnaires were collected, and the working group considered the feedback. Subsequent modifications were performed regarding relevance and rephrasing for clarification. The final registry form was then merged into the Stratum platform.

### Launching and implementing the national quality registry

A user manual was created, with explanatory text for each of the items on the registration form. To increase the user-friendliness, before nationwide implementation, a step-wise launching plan was devised. This process started with the introduction of the registry at the six largest clinics in Sweden for a duration of 3 months, during the third quarter of 2017. This period was followed by another phase of feed-back to identify any minor adjustments needed for the manual, the registration form, and the web-based platform. Thereafter, a nationwide launch was initiated in January 2018. All clinics in Sweden that performed orthognathic surgery where contacted (*n* = 21), informed about the registry, and invited to join. This personal contact, in combination with written information, was considered essential for achieving credibility. Furthermore, a contact person from the steering group was allocated to each of the clinics. A key issue was to assure the anonymity of individual surgeons. In parallel with the launching, educational efforts were planned to encourage confidence, achieve acceptance, and raise the awareness of the benefits of an NQR. These educational efforts included oral reports delivered at the annual meetings of The Swedish Association of Oral and Maxillofacial surgeons and the Swedish Dental Society.

### Ethical approval

The present study was reviewed and approved by the Swedish Ethics Review Board, Dnr 2020–05609. Recorded data were coded, and the management of data follows Swedish law and Swedish implementation of the EU Data Protection Directive 95/46/EC. According to Swedish governmental regulation patient’s inclusion in national registers is proceeded by information, which does not require written consent. Patients’ inclusion is voluntary and are free to be excluded upon active statement of this.

### Process, data, and analyses

For this study, we retrospectively audited data that were registered in the NROK in 2018–2019. The registry was started in November 2017, after approximately one and a half years of preparation. First, contact was made with one of the Swedish Registry Centers – there are six in the country. After an initial informative description of the process, a steering group was gathered, which consisted of six oral and maxillofacial surgeons, one orthodontist, and one anesthesia nurse, representing different geographic regions in the country. The next step was to learn from the existing quality registries. A digital platform, *Stratum*, which could be adjusted to our purposes, was identified at the Registry Center West in Gothenburg, and the steering group started working on the parameters that should be included. The registry was then approved by the Regional Council, Västra Götaland County.

By January 2018, 14 out of 21 centers that performed orthognathic surgery had joined the registry. By the end of 2019, this figure had increased to 19 participating clinics. This coverage included a total of 862 patients (413 men and 449 women) that had undergone orthognathic surgery. The inclusion criteria for treatment were: a confirmed diagnosis of a dentofacial deformity, patient willingness to undergo treatment, and medical fitness to undergo general anesthesia and surgery.

### Initial registration

Baseline data were based on the records documented on the day of surgery. The date of surgery and the patient’s age, sex, height, and weight were recorded. Next, anamnestic findings, the diagnosis, and the type of surgery were recorded. Then, the duration of surgery, the estimated amount of bleeding, and preoperative and intraoperative treatments were recorded.

## Results

### Coverage

The average coverage of the NROK was estimated to be 91% (*n* = 862, registered by the users) of the total number of orthognathic operations (*n* = 945) performed in Sweden during 2018–2019.

### General health status

In total, 84% (*n* = 724) of the patients were generally healthy at the time of surgery. The remaining 16% (*n* = 138) had one of the following conditions: autoimmune disease (2.7%, *n* = 23), psychiatric disorder (2.0%, *n* = 17), neuropsychiatric condition (3.2%, *n* = 28), metabolic disease (2.1%, *n* = 18), temporomandibular joint dysfunction (0.5%, *n* = 4), and other conditions (5.5%, *n* = 48). In the study population, 20.4% (*n* = 176) were taking some type of medication.

### Tobacco use

In total, only 5% (*n* = 43) of the patients were active smokers at the time of the operation. Most patients (89%) had never smoked, 3% (*n* = 26) had stopped smoking more than 3 months prior to the operation, and 3% (*n* = 26) had stopped within the 3-month period prior to surgery.

### Diagnosis

**Fig. 1:**
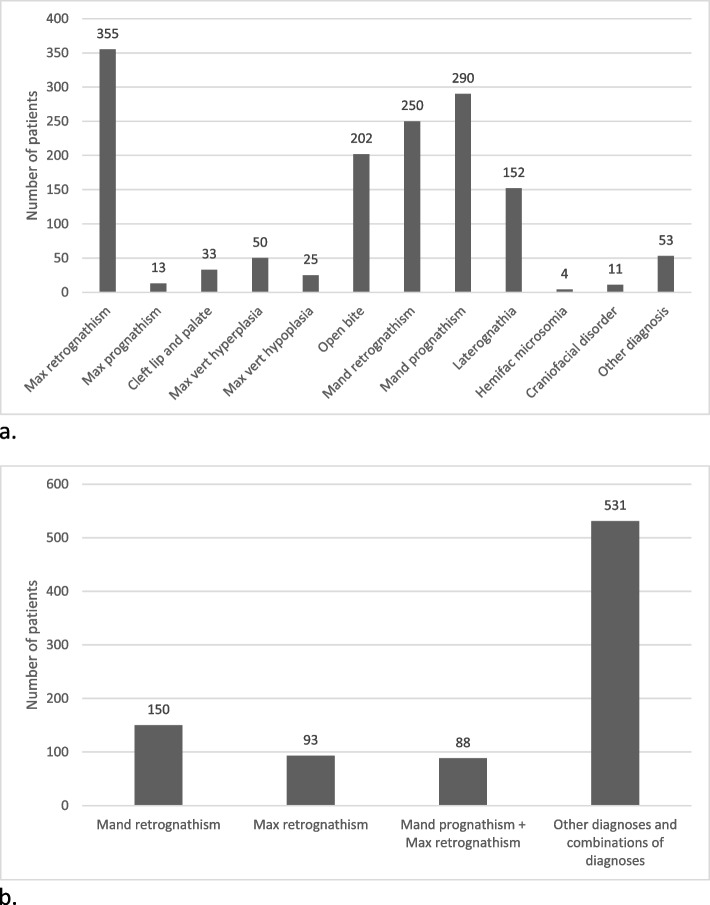
Display of diagnoses. 2a) shows the total number of diagnoses that were presented (some patients had more than one diagnosis) and 2b) shows the most common diagnoses separately (first two bars from left) and the most combinations of diagnoses (the two bars to the right). Abbreviations: Max, maxillary; vert, vertical; Mand, mandibular; Hemifac, hemifacial

Among the 862 included patients, 1438 dentofacial anomalies were diagnosed. The most common diagnoses were maxillary retrognathism (25%) and mandibular prognathism (20%) (Fig. 1a). When diagnoses were categorized as either upper- or lower-jaw involvements, the mandible was affected most frequently (38%, *n* = 540). A major proportion of patients (62%, *n* = 531) had more than one diagnosis, and the most common combination was concurrent mandibular prognathism and maxillary retrognathism. Among patients with a single diagnosis, retrognathism of either the mandible or maxilla was the most frequent anomaly (Fig. 1b). Craniofacial deformities were treated in 11 (1.3%) cases, and they were treated at two designated centers.

### Orthodontic treatment

Preoperative orthodontic treatment with fixed appliances was performed in 659 patients (76.5%). The other 203 patents were treated with either a surgery-first approach, (i.e., less than 3 months of orthodontic treatment before surgery), or surgery only (i.e., without any orthodontic treatment).

### Surgical treatment

**Fig. 2:**
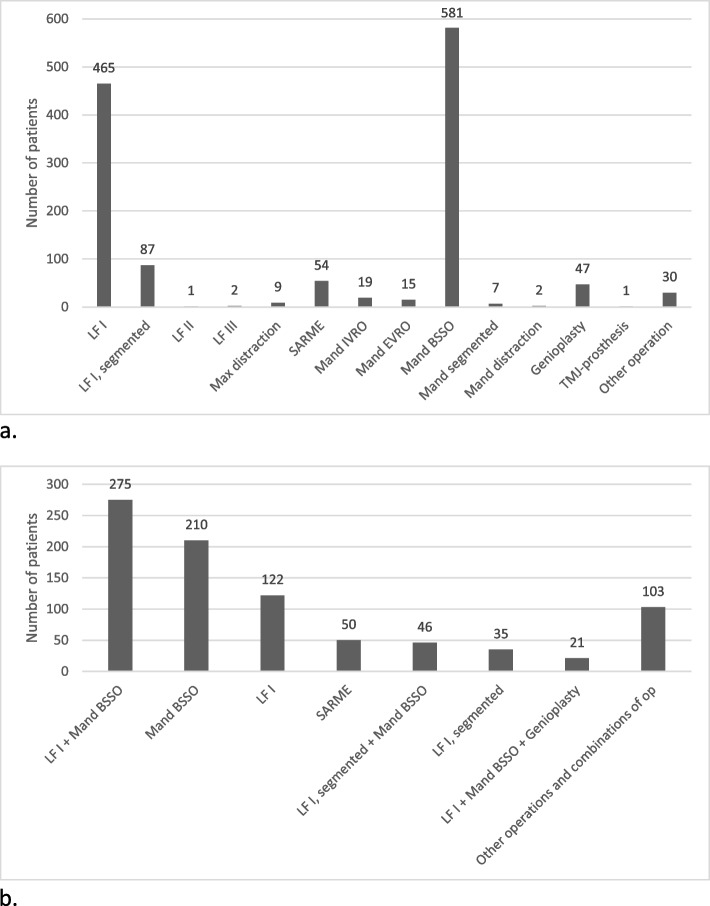
Illustration of surgical procedures. Figure 3 shows a) the total number of operations and b) the most common combinations of operations. Abbreviations: LF, Le Fort; Max, maxillary; SARME, surgical assisted rapid maxillary expansion; IVRO, intraoral vertical ramus osteotomy; EVRO, extraoral vertical ramus osteotomy; BSSO, bilateral sagittal split osteotomy; Mand, mandibular; TMJ, temporomandibular joint; op, operation

A total of 1320 surgical procedures were recorded in the registry. The two most frequent surgical procedures were the bilateral sagittal split osteotomy (BSSO, 44%) and Le Fort I osteotomy (35%; Fig. 2a). However, most patients received a combination of procedures (Fig. 2b). The most frequent combined surgical treatment was the Le Fort I combined with the BSSO (*n* = 275), and the second most common was the BSSO (*n* = 210) followed by the Le Fort I (*n* = 122). A less common technique was a mandibular setback, such as the intraoral vertical ramus osteotomy (IVRO) or the extraoral vertical ramus osteotomy (EVRO); this technique was performed in 34 patients (4%). A compensatory genioplasty was performed in 47 patients (*n* = 5%), most often in combination with a Le fort I osteotomy and BSSO.

**Fig. 3:**
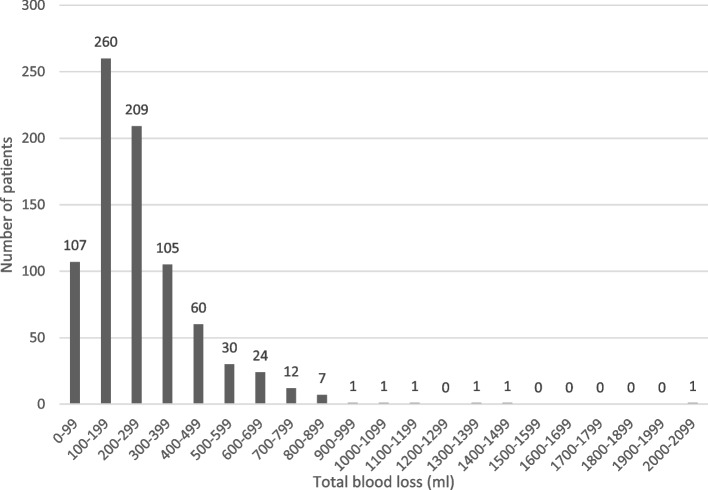
Blood loss in ml during surgery

Perioperative bleeding was routinely measured during surgery, and it was one of the quality parameters included in the registry. Perioperative bleeding was reported in 95% of patients (*n* = 820); 90% (*n* = 741) of patients had ≤500 ml blood loss, and 70% (*n* = 576) of patients had < 300 ml blood loss (Fig. 3). A bleeding complication, defined as > 1000 ml blood loss, was recorded in five cases (< 1%).

### Discharge, reoperations, and patient satisfaction

**Fig. 4:**
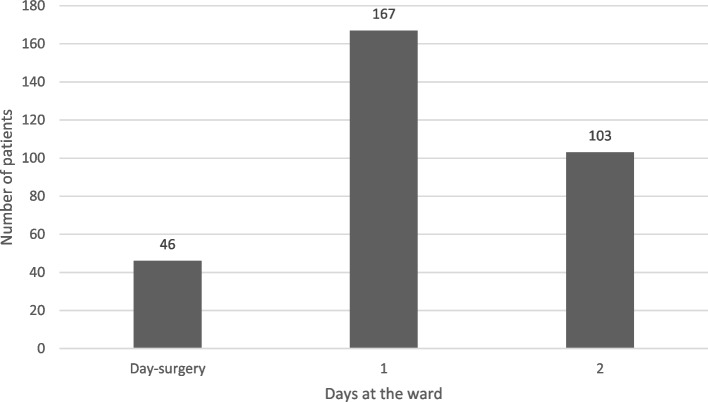
Illustration of length of hospital stay after orthognathic surgery

Most patients subjected to orthognathic surgery were admitted to the hospital. However, 46 (5%) patients underwent day surgery, where the patient could leave the hospital after fully recovering from anesthesia (Fig. 4). Among the in-patients, 270 (31%) were discharged from the hospital at 1–2 days after surgery. At follow up, 3.6% of the patients had undergone a reoperation. The majority of reoperations comprised plate removals, due to a postoperative infection. The treatment was rated satisfactory by 95% of patients at 6–24 months postoperatively.

## Discussion

Because NQRs collect and store patient data within the healthcare system, they are acknowledged as tools for improving health care for targeted patient groups and increasing scientific knowledge within the corresponding field. In Sweden, NQRs are well-established, and they are integrated into the healthcare system to allow open comparisons. Currently, there are over 100 NQRs with economic support from the government [35]. However, until 2017, there were no registries within the field of oral and maxillofacial surgery.

Most patients registered in the NROK were in their 20’s and generally healthy. This result was consistent with previous studies that described patients with facial deformities that required orthognathic surgery [1, 2, 6]. However, these patients often require surgical corrections with complex procedures that involve a substantial amount of soft and hard tissues in the face. Because most of these patients are otherwise healthy young adults, it was hypothesized that they might have much lower acceptance of complications and adverse outcomes, compared to patients that require other types of facial surgery, such as reconstructions due to facial trauma. For this type of question, the NQR can serve as an important tool for monitoring healthcare quality; its data can be analyzed to reveal both expected and unexpected risks. With increasing patient involvement in treatment planning, the ability to present data on in-house treatment results is likely to be increasingly important for the healthcare provider including the individual surgeon.

Coverage is an essential factor in the generalizability of NQR data. Since orthognathic surgery in Sweden is only performed under national health care insurance, clinics must register every surgical procedure to be economically compensated. The registered procedure codes are compiled in a database at the National Board of Social Affairs and Health, which enables reliable calculations of coverage rates of the NQR. Coverage is particularly important in cases of decentralized care, where many centers are involved. Decentralized care has the benefit of broad availability to patients. Considering orthognathic surgery, availability may be particularly important, due to long-term treatment collaborations between orthodontists and oral and maxillofacial surgeons. However, the potential drawbacks of a decentralized organization include insufficient patient volumes and difficulty in performing large-scale research. Adherence to an NQR can overcome these putative drawbacks by facilitating knowledge-sharing and comparisons of patient treatments and care between institutions; these advantages can lead to improved quality of care [35]. Because NROK was the first NQR in oral and maxillofacial surgery, education was considered necessary in a community of surgeons that were neither accustomed to nor experienced in the transparency provided by a registry. We found that an inclusive design process, where colleagues identified relevant parameters, was paramount to promoting broad acceptance. The coverage of NROK was 90% within only 2 years after launching. This coverage was better than initially anticipated, considering the decentralization of orthognathic care for patients in Sweden, the involvement of over 20 different centers, and the relative inexperience of the surgeons with NQRs. The high coverage rate indicated that Swedish oral and maxillofacial surgeons were truly interested in improving the quality of care in orthognathic surgery. A probable contributing factor was the user-friendly platform, which facilitated the planning, design, and launching of NROK. The NROK coverage was comparable to those of other established Swedish medical quality registries [35].

During the Covid-19 pandemic, there was an expected, tremendous drop in NROK registration, due to reallocation of healthcare resources to covid care. In the start-up period, post covid, there was a need for new information/re-motivation. To date, the registry is functioning, but for continued success, it is highly important for the NROK steering group to gain experience through close and regular contact with users.

Previously, smoking was found to be a significant risk factor for postoperative infections [36]. Smoking cessation was shown to impact post-operative complications, even when cessation was introduced as late as 4 weeks prior to surgery [37]. Among the patients registered in the NROK, 11% had either been smokers or were active smokers. Of these, 3% ceased smoking more than 3 months prior to surgery, and another 3% ceased smoking less than 3 months prior to surgery; thus, 5% of the registered patients were active smokers at the time of surgery. Interestingly, these rates were higher than the smoking rates of the total Swedish population, which was 6% in 2021, and 3% in the 16–29-year-old age group [38]. Thus, previous or active smoking was more common among the patients that underwent orthognathic surgery than in the general population, particularly in younger age groups. The reasons for this result, and the use of a pending surgical procedure as a motivation for smoking cessation, should be addressed in futures studies. This finding emphasized the importance of discussing with these patients the influence of tobacco use on the surgical outcome.

Most patients received preoperative orthodontic treatment, and the remaining patients were treated with the surgery-first approach or surgery only. Surgery-first or surgery only is increasingly advocated in select cases of orthognathic surgery, because it has the advantage of reducing the treatment time [39]. The surgery-first approach was shown to contribute to better oral health-related quality of life in patients with dentofacial deformities, both in the short term and in the long term [40]. In the current study, patient satisfaction was 95% at last follow-up after surgery. This result requires confirmation in a larger cohort with sufficient statistical power.

In the current study, 40% of the treatments included bimaxillary surgery. The decision between bimaxillary and single-jaw surgery is complex; several factors must be considered, including the magnitude of the change and the relationship between the maxilla and the mandible. One important aspect of the post-surgical outcome is stability. A previous study compared skeletal and dental relapses after either a single-jaw or bimaxillary surgery for a correcting a skeletal class III deformity. They found that the single-jaw procedure could lead to less stability, and consequently, it was associated with more frequent skeletal relapses [41]. In contrast, a recent systematic review concluded that the single-jaw and two-jaw procedures for correcting class III deformities provided comparable skeletal outcomes [42].

In the present study, a BSSO, which enabled movement in all directions, was the most common (93%) surgical procedure in the mandible. In contrast, IVROs and EVROs, which only allowed setbacks, was performed in 4% of the cases. Although BSSO allows free post-surgical mandible movement, it carries a relatively higher risk of neurosensory disturbances in the inferior alveolar nerve, compared to IVRO or EVRO [43]. Based on the NROK data, we concluded that, in considering which surgical technique to choose, the expected discomfort of 4 weeks intermaxillary fixation outweighed the higher risk of neurosensory disturbance. However, the registry data did not provide clues to whether this choice was made by the surgeon or the patient. According to Swedish guidelines, patient involvement is required in the choice of treatment.

The safety aspects covered in the registry were perioperative bleeding, length of hospital stay, reoperations, and neurosensory disturbances at last follow-up. As expected, orthognathic surgery was considered safe, in terms of bleeding, which was typically well below the level of considering a blood transfusion. Less than 1% of the patients reported more than 1000 ml of blood loss. Most patients could be discharged after one overnight admission. This result was consistent with other reports from the United Kingdom and Denmark [44, 45]. The short hospital stay suggested that it may be possible to increase outpatient surgery in selected cases. No financial analysis has been made so far but all included data from NROK can be used for analysis of cost benefit for example during the comparacy of day patients and in-patients. The included data from orthognathic surgical procedures in Sweden serve as database from where, new research in the orthognathic surgery area can be evaluated.

## Conclusion

In conclusion, this study described a novel quality registry that functioned with good adherence and coverage. The availability of these data will strengthen decision-making among clinicians. Our findings suggested that orthognathic surgery was safe and predictable, and improved the quality of life for the patients.

## Data Availability

All data in the NROK register is available for studies after application to the steering group for NROK (contact information homepage: https://nrok.registercentrum.se/kontakt/kontakt/p/B1SOVye8Q) together with an approved ethic approval to the Swedish Ethics Review Board.

## Abbreviations

NQR: National quality registry
NROK: Swedish national registry for orthognathic surgery
BSSO: Bilateral sagittal split osteotomy
IVRO: Intraoral vertical ramus osteotomy
EVRO: Extraoral vertical ramus osteotomy
